# From gut to liver: unveiling the differences of intestinal microbiota in NAFL and NASH patients

**DOI:** 10.3389/fmicb.2024.1366744

**Published:** 2024-04-04

**Authors:** Furong Huang, Bo Lyu, Fanci Xie, Fang Li, Yufeng Xing, Zhiyi Han, Jianping Lai, Jinmin Ma, Yuanqiang Zou, Hua Zeng, Zhe Xu, Pan Gao, Yonglun Luo, Lars Bolund, Guangdong Tong, Xu Fengping

**Affiliations:** ^1^Faculty of Chinese Medicine and State Key Laboratory of Quality Research in Chinese Medicine, Macau University of Science and Technology, Macau, China; ^2^Department of Hepatology, Shenzhen Traditional Chinese Medicine Hospital, Shenzhen, China; ^3^Department of Sanming Project of Medicine in Shenzhen, Shenzhen Traditional Chinese Medicine Hospital, Shenzhen, China; ^4^College of Life Sciences, University of Chinese Academy of Sciences, Beijing, China; ^5^BGI Cell, Shenzhen, China; ^6^The Fourth Clinical Medical College of Guangzhou University of Chinese Medicine, Shenzhen, China; ^7^People's Hospital of Longhua, Shenzhen, China; ^8^BGI, Shenzhen, China; ^9^Qingdao-Europe Advanced Institute for Life Sciences, BGI Research, Qingdao, China; ^10^Lars Bolund Institute of Regenerative Medicine, BGI-Qingdao, BGI Research, Qingdao, China; ^11^Department of Infectious Diseases, Shenzhen Traditional Chinese Medicine Hospital, Shenzhen, China

**Keywords:** NAFLD, gut microbiome, NASH, 16S rRNA sequencing, NAFL

## Abstract

Non-alcoholic fatty liver disease (NAFLD) is increasingly recognized for its global prevalence and potential progression to more severe liver diseases such as non-alcoholic steatohepatitis (NASH). The gut microbiota plays a pivotal role in the pathogenesis of NAFLD, yet the detailed characteristics and ecological alterations of gut microbial communities during the progression from non-alcoholic fatty liver (NAFL) to NASH remain poorly understood. Methods: In this study, we conducted a comparative analysis of gut microbiota composition in individuals with NAFL and NASH to elucidate differences and characteristics. We utilized 16S rRNA sequencing to compare the intestinal gut microbiota among a healthy control group (65 cases), NAFL group (64 cases), and NASH group (53 cases). Random forest machine learning and database validation methods were employed to analyze the data. Results: Our findings indicate a significant decrease in the diversity of intestinal flora during the progression of NAFLD (*p* < 0.05). At the phylum level, high abundances of Bacteroidetes and Fusobacteria were observed in both NAFL and NASH patients, whereas Firmicutes were less abundant. At the genus level, a significant decrease in Prevotella expression was seen in the NAFL group (AUC 0.738), whereas an increase in the combination of Megamonas and Fusobacterium was noted in the NASH group (AUC 0.769). Furthermore, KEGG pathway analysis highlighted significant disturbances in various types of glucose metabolism pathways in the NASH group compared to the NAFL group, as well as notably compromised flavonoid and flavonol biosynthesis functions. The study uncovers distinct microbiota characteristics and microecological changes within the gut during the transition from NAFL to NASH, providing insights that could facilitate the discovery of novel biomarkers and therapeutic targets for NAFLD.

## 1 Introduction

Non-alcoholic fatty liver disease (NAFLD) has emerged as a prominent global health challenge in the field of hepatology, characterized by abnormal accumulation of lipids in the liver, including non-alcoholic fatty liver (NAFL) and non-alcoholic steatohepatitis (NASH; Scavo et al., 2022; Huang et al., 2023; Ren et al., 2023). The latter often progresses to cirrhosis, liver failure, and hepatocellular carcinoma (HCC), making it the leading cause of liver transplantation (Davis et al., 2019; Huang et al., 2021). Despite advancements in understanding the pathogenesis and therapeutic approaches for NAFLD (Sun et al., 2019), significant challenges remain in the early diagnosis and treatment of the disease.

The gut microbiota, which refers to the microbial community residing within the gastrointestinal tract, plays a crucial role in host digestion, immunity, and metabolism. Dysbiosis, characterized by unhealthy alterations in the normal bacterial ecology, pervades the entire pathological continuum of NAFLD, encompassing conditions from simple steatosis to NASH, and extending through to advanced fibrosis, culminating in cirrhosis and hepatocellular carcinoma (Hoyles et al., 2018; Javdan et al., 2020; Mohr et al., 2020).

The foundational study by Demir et al. presented groundbreaking evidence supporting a causal relationship between the gut microbiota and the pathogenesis of NAFLD, as demonstrated by mouse models and fecal transplant experiments (Demir et al., 2022). The findings revealed an increase in two distinct bacterial species (Trichophylidae bacteria 609 and Barnesiella) among mice exhibiting steatosis (Jin et al., 2022). However, significant disparities were observed in terms of composition, predominant genera, as well as abundance levels for specific genera and species when comparing the gut microbiota profiles between mice and humans (Javdan et al., 2020; Mohr et al., 2020). Therefore, it is imperative to detect and differentiate the disparities between gut microbiota and NAFLD in human subjects. In recent years, numerous studies have concentrated on the characteristics of gut microbiota in human patients with steatosis, NASH, or related cirrhosis. Current research has demonstrated an increase in Proteus (Raman et al., 2013; Shen et al., 2017; Hoyles et al., 2018; Loomba et al., 2019) and Enterobacterium (Hoyles et al., 2018) within NAFLD patients. Conversely, Riederia (Zhu et al., 2013; Del et al., 2017) and Ruminococcus exhibited a reduction at the genus level (Zhu et al., 2013; Del et al., 2017). Specifically, Escherichia (Zhu et al., 2013; Hoyles et al., 2018) Doxella (Zhu et al., 2013; Del et al., 2017), and Gastrobacillus (Zhu et al., 2013; Del et al., 2017) displayed an upward trend. On the other hand, anaerobic sporococcus (Wang et al., 2016; Roeb, 2022), Bacillus faecalis (Wang et al., 2016; Hoyles et al., 2018), Bacillus (Zhu et al., 2013; Hoyles et al., 2018), Bacillus faecalis (Zhu et al., 2013; Roeb, 2022), and Prevotella (Boursier et al., 2016; Hoyles et al., 2018) all showed a downward trajectory. The microbial signatures observed in NASH patients were similar to those found in NAFLD when compared to healthy individuals serving as controls. However, these findings exhibited variations attributed to disparities in cohort demographics, including but not limited to sex, ethnicity, severity of liver disease stage, presence or absence of type 2 diabetes mellitus and BMI, as well as the patient population being either pediatric or adult. An additional factor that could complicate the interpretation of changes in microbiota composition in NAFLD is sarcopenia. Xiangya study has found that the relative abundance of six species (*Desulfovibrio piger*, *Clostridium symbiosum*, Hungatella effluvii, *Bacteroides fluxus*, Absiella innocuum, and *Clostridium citroniae*) was also positively associated with the severity of sarcopenia (Wang et al., 2022). Sarcopenia is a common complication of various chronic diseases (Tarantino et al., 2023), yet its specific molecular mechanisms remain unclear. Sarcopenia’s close relationship with the gut microbiome has been documented in studies by Nikkhah et al. (2023) and Ren et al. (2021) and the prevalence of sarcopenia in patients with NAFLD has been noted by Cai et al. (2020). Additionally, discrepancies in gut microbiota composition were influenced by factors such as obesity-related metabolic diseases and the sequencing techniques employed for gut microbiota analysis. Consequently, certain contemporary studies even present contradictory trends when compared to the aforementioned results (Kim et al., 2021; Galli et al., 2022; Girdhar et al., 2023).

Therefore, in this study, 16S rRNA sequencing technology was employed to systematically analyze intestinal samples from Chinese individuals pathologically diagnosed with NAFL and NASH, and compare them with healthy controls. By investigating the variations in intestinal microflora composition at different stages of NAFLD and further exploring the correlation between alterations in the abundance of dominant strains of intestinal microflora among patients with distinct disease stages such as NAFL and NASH, functional analysis and mechanistic studies will contribute to comprehending the specific role of these microflora in NAFLD progression. The findings from this investigation are anticipated to offer novel biomarkers and therapeutic targets for diagnosing and treating NASH. Through understanding the association between gut microbiota and NASH, we can enhance our comprehension of NASH pathogenesis while developing new strategies and approaches for its prevention and treatment.

## 2 Materials and methods

### 2.1 Study design

This study aimed to investigate the role of gut microbiome in patients with NAFL or NASH through 16S rRNA gene amplicon sequencing. Patients diagnosed with NAFL or NASH, as well as a normal control population, were recruited for this study. Detailed demographics are provided in Table 1. Stool samples from the patients were collected and immediately stored in −80°C at the time of each research visit. DNA extraction of stools, amplification of bacterial 16S rRNA genes, cloning, and sequencing of polymerase chain reaction (PCR) products were performed at the BGI-Shenzhen laboratory. This study was approved by Ethics Committee of Shenzhen Hospital of Traditional Chinese Medicine (ethical approval number: K2017-024), all the participants who agreed to serve as fecal donors provided written informed consent, in accordance with national legislation and the Code of Ethical Principles for Medical Research Involving Human Subjects of the World Medical Association (Declaration of Helsinki).

**Table 1:** Characteristics of each group in detail, including clinical, metabolic, biochemical and histological profiles (mean, mean ± sd).

Variables (mean, sd)	Control	NAFL	NASH	FDR* (NASH vs. NAFL)
	*N* = 65	*N* = 64	*N* = 53	
Age, years	37.03 (9.19)	37.83 (9.29)	34.79 (8.32)	0.070076
Female sex, n (%)	35 (0.54)	31 (0.48)	5 (0.09)	0.000000
Smoking, n (%)				
Current smoker	26 (0.4)	25 (0.39)	9 (0.17)	0.000000
Ex-smoker	2 (0.03)	2 (0.03)	6 (0.11)	0.000000
Non-smoker	37 (0.57)	37 (0.58)	38 (0.72)	0.792146
BMI, kg/m2	22.76 (2.84)	29.26 (3.60)	29.44 (4.56)	0.000037
WC, cm	76.46 (12.57)	89.81 (10.83)	98.11 (7.70)	0.000342
CAP, dB/m	213.25 (18.84)	304.64 (35.16)	325.38 (35.84)	0.000757
E, kPa	4.31 (1.22)	5.12 (1.35)	9.25 (11.87)	0.000000
ALT, U/L	16.46 (7.00)	22.10 (7.23)	76.17 (34.50)	0.000000
AST, U/L	20.96 (8.49)	19.18 (3.89)	40.22 (15.45)	0.000000
GGT, U/L	17.94 (17.00)	27.48 (17.49)	71.45 (47.93)	0.000091
ALP, U/L	54.75 (14.00)	62.10 (16.68)	73.34 (14.43)	0.278399
TBIL, umol/L	15.58 (5.88)	15.01 (8.71)	16.39 (5.13)	0.21574
DBIL, umol/L	3.49 (1.84)	3.16 (1.82)	3.57 (1.64)	0.441257
IBIL, umol/L	12.09 (4.72)	12.03 (7.22)	12.82 (3.74)	0.003631
ALB, g/L	47.93 (2.39)	48.87 (2.30)	50.43 (3.81)	0.496058
PLT, %	258.31 (53.98)	284.52 (66.26)	273.00 (139.86)	0.000006
AFP, mg/L	2.83 (1.78)	3.23 (1.90)	1.68 (1.68)	0.000005
CR, mmol/L	68.57 (15.43)	66.59 (14.25)	78.92 (12.10)	0.000006
UA, umol/L	325.32 (84.63)	382.16 (80.30)	453.94 (83.03)	0.91268
eGFR	106.91 (13.25)	102.24 (22.07)	101.88 (16.32)	0.019299
TG, mmol/L	1.11 (0.54)	1.77 (1.73)	2.04 (0.77)	0.047391
TC, mmol/L	4.35 (0.76)	4.72 (0.99)	5.05 (0.91)	0.300345
LDL, mmol/L	6.73 (32.17)	3.09 (0.95)	3.28 (1.01)	0.818905
FBG, mmol/L	4.42 (0.87)	5.15 (0.56)	5.18 (0.63)	0.314147
HBAlc, %	5.62 (0.31)	5.70 (0.33)	5.76 (0.32)	0.004465
FBC, nmol/L	1.52 (0.63)	2.36 (0.98)	2.91 (1.41)	0.005862
FBI, pmol/L	46.85 (27.25)	86.17 (44.70)	116.34 (91.35)	0.054905
SF, umol/L	18.31 (6.98)	18.28 (6.30)	20.51 (6.10)	0.000000
FER, nmol/L	123.58 (113.66)	145.72 (117.95)	328.30 (211.70)	0.144514
CER, g/L	0.14 (0.03)	0.17 (0.04)	0.16 (0.04)	0.000000
AST/ALT	1.37 (0.43)	0.93 (0.24)	0.57 (0.21)	0.000000
SAF score	–	1.52 (0.31)	4.81 (0.74)	0.005000
HOMA-IR	1.37 (0.91)	2.75 (1.48)	3.71 (2.84)	0.005000
TyG	7.4 (0.39)	7.52 (0.64)	7.65 (0.32)	0.000000

*By Mann–Whitney test. HOMA-IR calculation formula: FPG (mmol/L) × FBI (μU/mL)/22.5. TyG calculation formula: ln [TG (mg/dl) × FBG (mg/dl)]/2.

### 2.2 Patient selection

#### 2.2.1 Study participants

**Figure 1:**
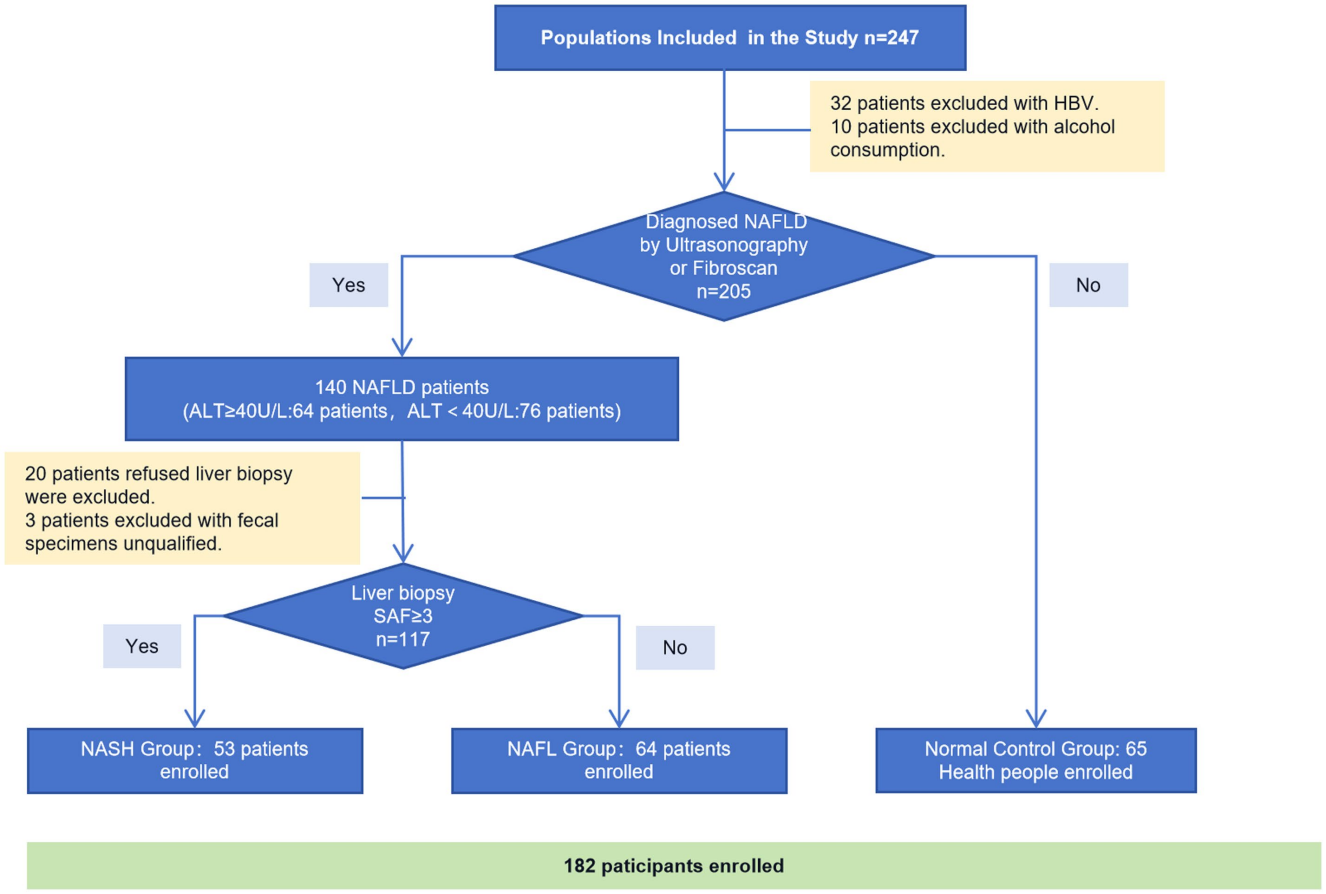
Consort flow diagram of study participants.

Participants were recruited from the Department of Hepatology at Shenzhen Traditional Chinese Medicine Hospital between December 2018 and December 2019. A total of 237 individuals underwent primary screening, with 32 patients excluded due to HBV infection and 10 patients excluded due to alcohol consumption. Among the remaining 205 subjects, ultrasound or Fibroscan was performed to diagnose NAFLD and categorize them into 140 NAFLD patients and 65 normal controls. 64 patients out of the 140 with NAFLD showed abnormal ALT levels (ALT≥40 U/L). All of these 140 NAFLD patients were advised to undergo a liver biopsy and provide fecal samples; however, 20 of them refused the liver biopsy and three patients had unqualified fecal specimens. Finally, a total of 53 NASH patients with confirmed biopsies, along with 64 biopsy-proven NAFLD patients and 65 healthy controls were enrolled (Figure 1).

#### 2.2.2 Inclusion criteria

Inclusion criteria were: (1) Presence of hepatic steatosis confirmed by liver imaging techniques such as ultrasonography or fibroscan; (2) Absence of any concurrent chronic liver diseases including viral hepatitis or autoimmune hepatitis; (3) Alcohol consumption below 20 g/day.

#### 2.2.3 Liver biopsy and histopathology

The percutaneous liver biopsy was performed under ultrasound guidance following local anesthesia. A 16-G Tru-Cut biopsy needle (C.R. Bard Inc., Covington, United States) was inserted into the liver parenchyma to obtain an adequate amount of liver tissue containing a minimum of 10 portal tracts with an approximate length of 2.0 cm for precise diagnostic purposes. The obtained specimens were promptly fixed, embedded in paraffin, and stained with hematoxylin–eosin (HE). Subsequently, all specimens were sent to the Department of Pathology at Shenzhen Traditional Chinese Medicine Hospital for evaluation. Liver pathological images were diagnosed by two experienced pathologists who were blinded to the TE (CAP) value and color Doppler ultrasound scans. The SAF score was employed to semi-quantify each individual feature of NAFLD, encompassing steatosis, inflammatory activity, and fibrosis, in accordance with the SAF (0–7) scoring system (Bedossa et al., 2012). The classification of NAFLD comprises two subtypes: NAFL and NASH. The scoring criteria encompass hepatocyte steatosis (0–3), balloon-like degeneration (0–2), and lobular inflammation (0–2). NASH is diagnosed when both balloon-like degeneration and lobular inflammation coexist, or if the total score on each scale is ≥3; otherwise, NAFL is diagnosed.

#### 2.2.4 Genomics DNA extraction

DNA was extracted from fecal samples using the MagPure Stool DNA kit B (Magen, China) according to the provided protocol. Subsequently, the DNA was quantified utilizing a Qubit Fluorometer with the Qubit^®^ dsDNA BR Assay kit (Invitrogen, China), and its quality was assessed by electrophoresis on a 1% agarose gel.

#### 2.2.5 Library construction

Variable region V4 of bacterial 16S rRNA gene was amplified with degenerated PCR primers, 515F (5’-GTGCCAGCMGCCGCGGTAA-3′) and 806R (5’-GGACTACHVGGGTWTCTAAT-3′). The forward and reverse primers were both modified with Illumina adapter, pad, and linker sequences. PCR enrichment was conducted in a 50 μL reaction system containing 30 ng of template DNA using the following cycling conditions: pre-denaturation at 95°C for 3 min, followed by 30 amplification cycles consisting of denaturation at 95°C for 45 s, annealing at 56°C for 45 s, and extension at 72°C for 45 s. A final extension step was performed in 72°C for a duration of 10 min. Subsequently, the PCR products were purified using Agencourt AMPure XP beads and eluted in Elution buffer. The quality assessment of libraries was conducted using the Agilent Technologies’ bioanalyzer model number 2100. Once validated, these libraries were subjected to sequencing on Illumina HiSeq2500 platform (BGI, Shenzhen, China) following the standard protocols provided by Illumina. This resulted in the generation of paired-end reads with a length of 2 × 250 bp.

#### 2.2.6 Sequencing and bioinformatics analysis

The raw reads were subjected to adaptor removal, as well as the elimination of low-quality and ambiguous bases. Subsequently, the Fast Length Adjustment of Short reads program (FLASH, v1.2.11) was employed to merge paired-end reads with tags in order to obtain the final tags.

The tags were denoised into operational taxonomic units (OTUs) using two methods: unoise3 with VSEARCH (v2.22.1; Rognes et al., 2016), and DADA2 with QIIME2 v2023.2 (Bolyen et al., 2019). Chimera sequences were identified by comparing them to the gold database Silva (v123; Quast et al., 2012) using VSEARCH. Subsequently, an OTU feature table was generated for further analysis using VSEARCH (v2.22.1). Taxonomic classification of the OTU representative sequences was performed with Silva v123 (Pelin et al., 2013), employing a minimum confidence threshold of 0.6, and trained on the Greengenes database v13_8 through QIIME2(v2023.2; Yokono et al., 2018). Finally, USEARCH global (Edgar, 2010) was utilized to compare all tags back to the OTUs in order to obtain an abundance statistics table for each sample.

The estimation of alpha and beta diversity was conducted using USEARCH (v11.0.667; Edgar, 2010) and QIIME2 (v2023.2) at the OTU level, respectively. The visualization of diversity analysis was generated using the R package “amplicon” (Labun et al., 2019), while the Chord diagram illustrating different classification levels was created using the R package “circlize” (Gu et al., 2014).

Differential analysis was performed using the “edgeR” (Chen et al., 2016) package and STAMP (Parks et al., 2014). Volcano plots and Manhattan plots were generated using the “ggplot2” (Ginestet, 2011) package. LDA analysis was conducted using LEfSe (Zhang et al., 2013). A false discovery rate (FDR) of 0.05 and an LDA score greater than 2 were used as indicators of statistical significance. KEGG function prediction was carried out using the PICRUSt (Wilkinson et al., 2018) and PICRUSt2 (Douglas et al., 2020) software. Organism Level Microbiome Phenotypes were predicted using BugBase (Ward et al., 2017) software. Significant species of function were determined by R (v4.2.3), based on Wilcox-test or Kruskal-test.

#### 2.2.7 Statistical analysis

Because the data did not all satisfy the normal distribution, the Mann–Whitney test was used to test each clinical data. The statistical test of negative binomial distribution was used to analyze OTU differences by edgeR. The Wilcoxon rank sum test was used to analyze species differences between the two groups. LEfSe analysis firstly used the non-parametric Kruskal-Wallis rank sum test to detect species with significant differences between different groups in multiple groups of samples, and then used the Wilcoxon rank sum test for groups of significantly different species to analyze the differences between groups. Finally, linear discriminant analysis (LDA) was used to reduce the dimensionality of the data to evaluate the influence of species with significant differences. In the analysis of metabolic pathways, Welch’s t-test was used to compare the two groups. Data sets that involved more than two groups were assessed by one-way ANOVA followed by Tukey–Kramer *post-hoc* tests. *p* < 0.05 was considered statistically significant and Story false discovery rate (FDR) of 0.05 was used as indicators of statistical significance.

## 3 Results

### 3.1 Clinical characteristics of NAFLD population

This study included 64 patients diagnosed with NAFL and 53 patients diagnosed with NASH based on pathological examination, as well as 65 individuals without NAFLD serving as controls, according to the predefined inclusion criteria. The comprehensive characteristics of each group, encompassing clinical, metabolic, biochemical, and histological profiles, are presented in Table 1. Most of the clinical indices such as BMI, CAP, E, ALT, AST, GGT, ALP, UA, etc. showed significant variance. The values of CAP, ALT, E, HOMA-IR and other clinical indices gradually increased in the control, NAFL and NASH groups, and the indices in the NAFL and NASH groups were mostly higher than the normal values of liver function tests.

### 3.2 Species diversity of the gut microbiome in groups

**Figure 2:**
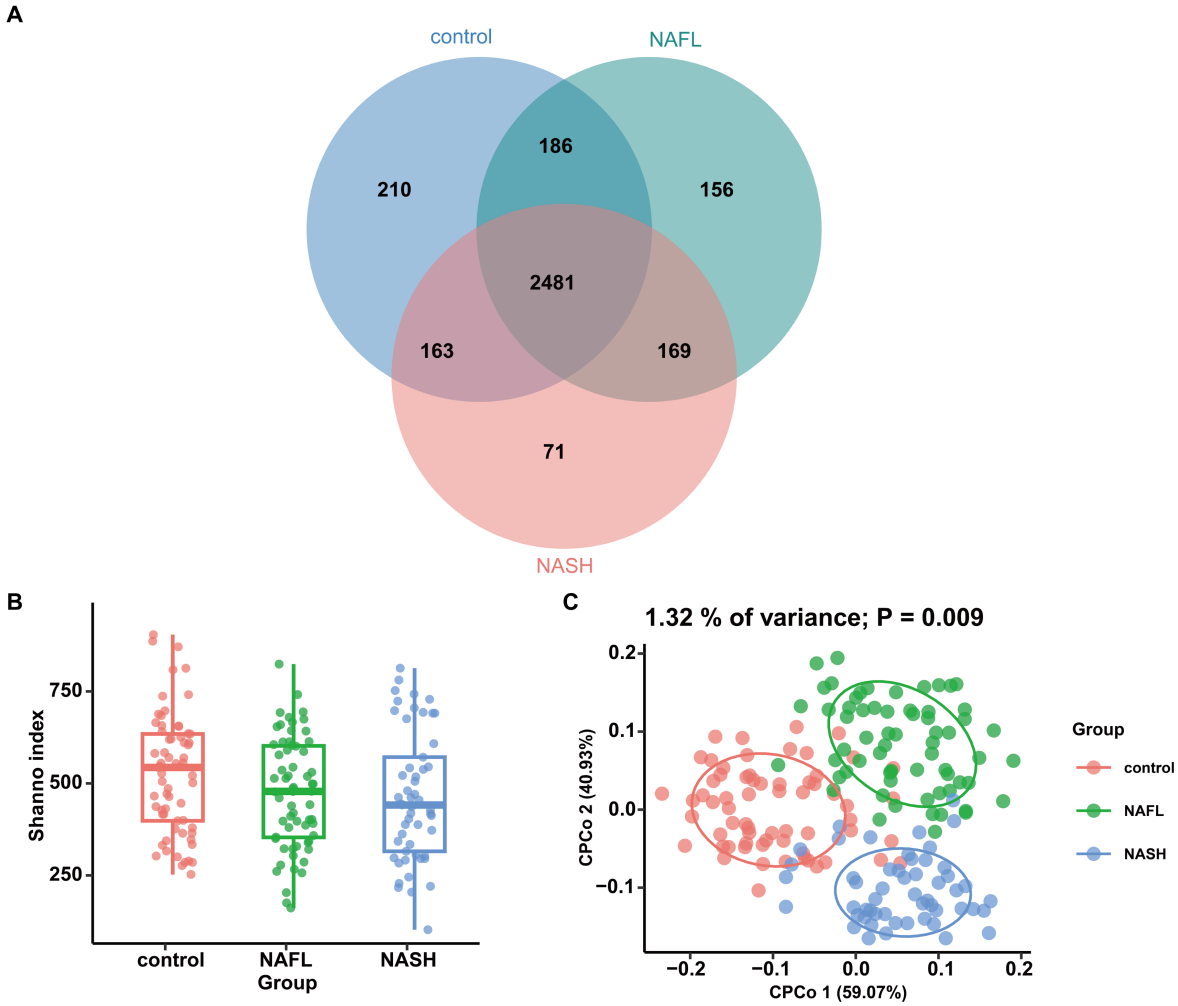
Comparisons of the alpha diversity and beta diversity about control, NAFL and NASH patients. **(A)** Venn diagram showing overlaps of the operational taxonomic units (OTUs) in the two groups (Shannon index). **(B)** Alpha diversity of the three groups, as quantified by diversity indices (Shannon, *p* < 0.05). **(C)** Principal coordinate analysis (CPCoA) plots of bacterial beta diversity of the three groups based on Euclidean. (*p* = 0.009).

In microbiome sequencing, the UNOISE3 algorithm yielded a total of 13,686 operational taxonomic units (OTUs). A sparse profile based on observed species indicates that the sequencing data is sufficient for detecting all species present in the samples (Supplementary Figure S1). To assess differences in bacterial diversity among the three groups, sequence alignment was performed to estimate both alpha and beta diversity. Our study revealed significant differences in α diversity between the NAFL group, NASH group, and control group (Shannon index, *p* < 0.05; Figure 2B). Beta diversity was subsequently evaluated using CPCoA rankings (Figure 2C), revealing a significant variation in species composition across the three groups (variance: 1.32%; *p* = 0.009). The Venn diagram demonstrated that there was a total of 2,481 OTUs shared among all three groups, with differentially expressed OTUs numbering at 210 for the control group, 156 for NAFL, and 71 for NASH. Furthermore, the number of group-specific taxa decreased from healthy control patients to NAFL patients to NASH patients (Figure 2A).

### 3.3 Differences in the taxonomic composition in groups

**Figure 3:**
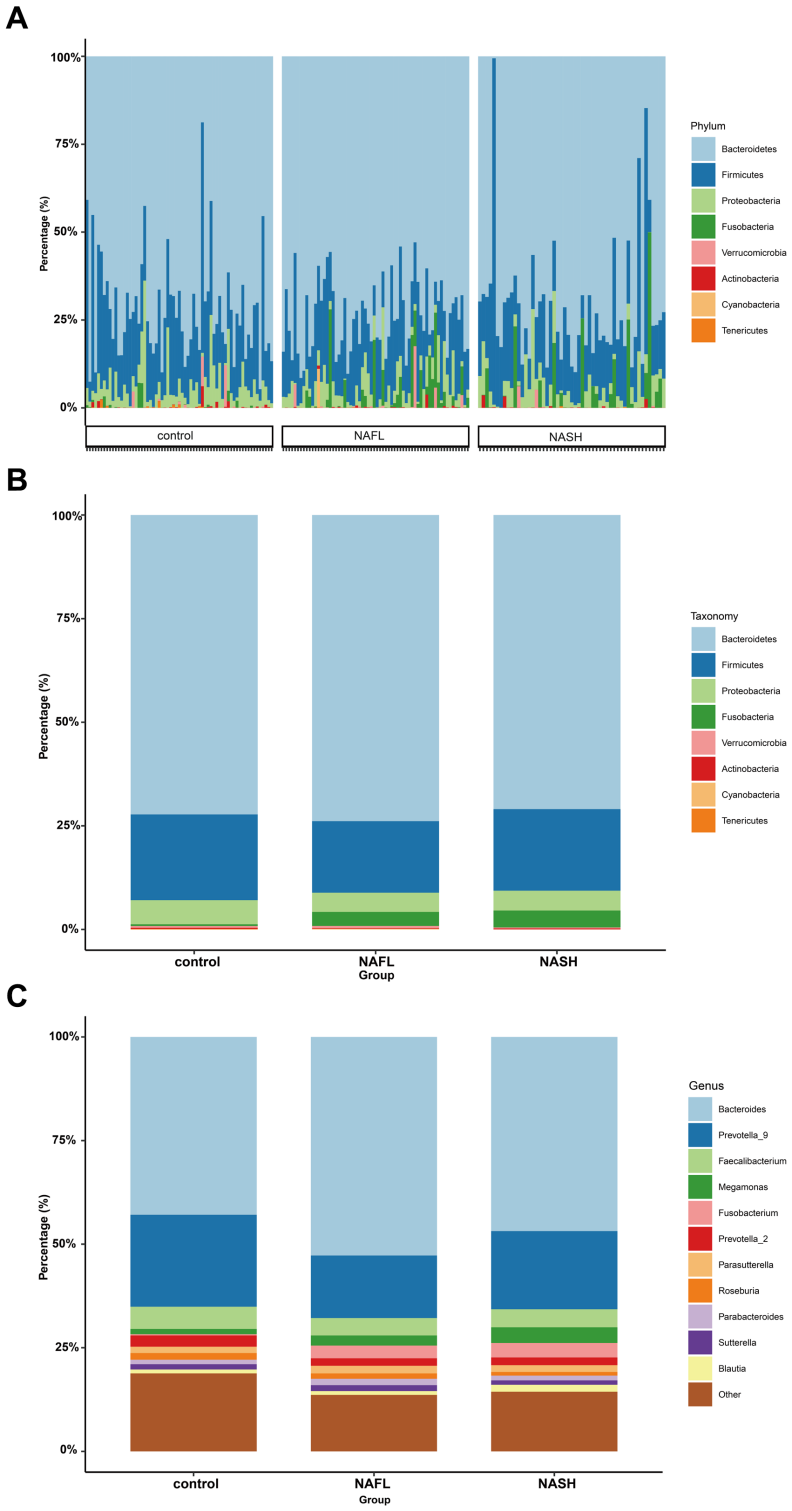
Comparisons of the taxonomic classifications of control, NAFL, NASH group. **(A)** Phyla composition for each sample. The species composition of all 182 samples is displayed, revealing significant variations in both the species distribution and relative abundance among each sample. Consequently, subsequent investigations can selectively focus on a few specific samples for comprehensive data analysis and clinical examination. *p* < 0.05. **(B)** Top 8 phyla in the three groups. *p* < 0.05. In the three groups, despite variations in the relative abundance of species, the phyla Bacteroidetes and Firmicutes consistently represented the largest fractions. This suggests that regardless of the health status—spanning from the healthy individuals, through those with NAFL, to patients with NASH—the key constituents of the gut microbiome remain fundamentally stable, without extreme shifts from dominance to depletion. **(C)** Top 11 genera in the three groups. *p* < 0.05. Upon refining the analysis down to the genus level, we discerned more defined taxonomic categories, and the distinctions between species became increasingly conspicuous. This enables the identification of potentially pathogenic bacteria associated with conditions such as NAFL or NASH. Such genus-level differential analysis is instrumental in establishing a foundation for clinical diagnosis, informing treatment strategies, and guiding preventative measures.

**Figure 4:**
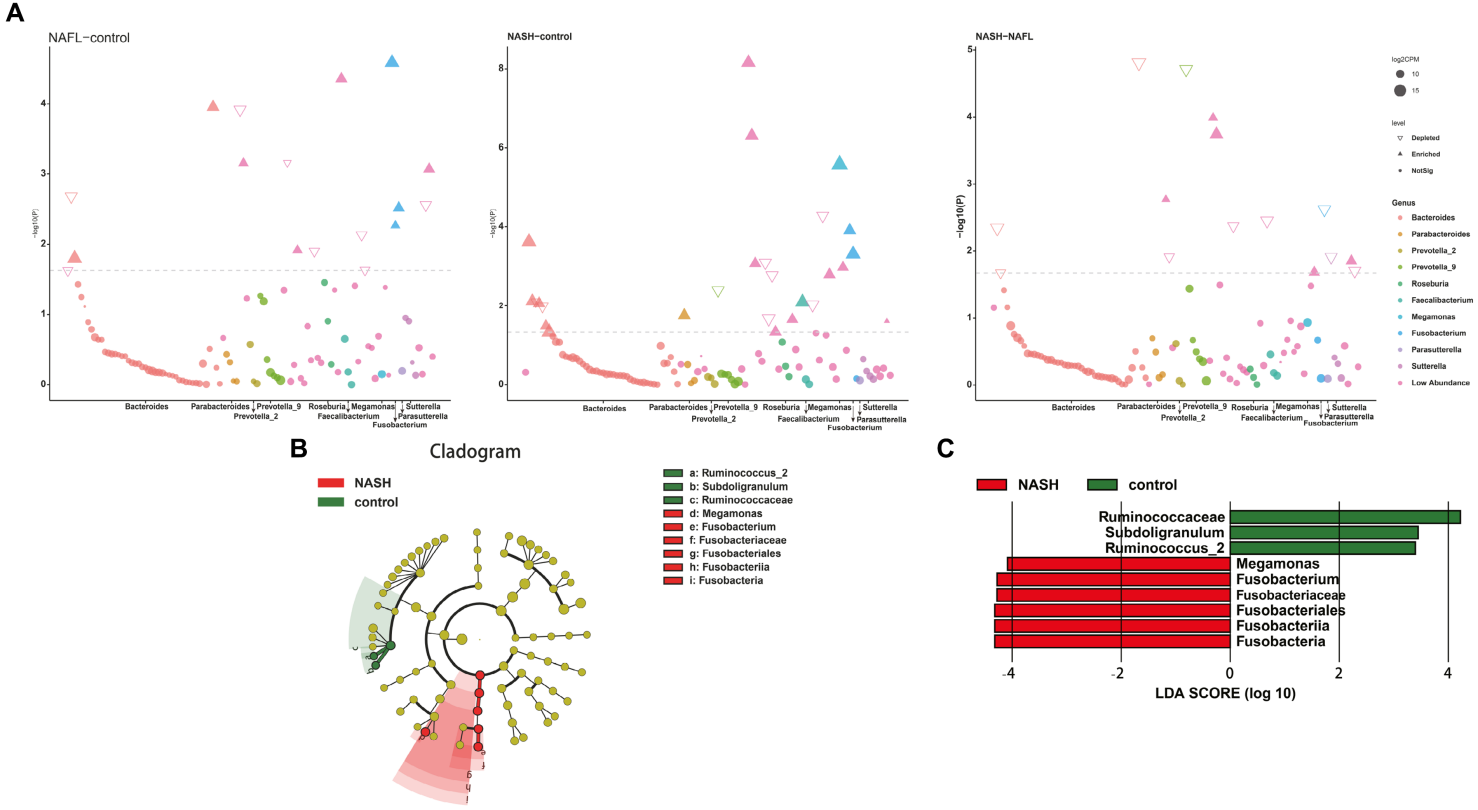
Bacterial biomarkers identified with the edgeR and linear discriminant analysis effect size (LEfSe) algorithm. **(A)** The results of the differential analysis of 10 genera including Bacteroides, Parabacteroides, Prevotella_2, Prevotella_9, Roseburia, Faecalibacterium, Megamonas, Fusobacterium, Parasutterella, Sutterella are presented. Manhattan plots for analysis of genus differences between NAFL and control groups (Figure 4A, left; *p* < 0.05). Bacteroides and Fusobacterium were significantly expressed in NAFL group compared with the control group. Manhattan plots for analysis of genus differences between NASH and control groups (Figure 4A, middle; *p* < 0.05). Faecalibacterium and Fusobacterium were significantly expressed in NASH group compared with the control group. Manhattan plots for analysis of genus differences between NASH and NAFL groups (Figure 4A,right; *p* < 0.05). Bacteroides was significantly decreased in NASH group compared with the NAFL group. **(B)** Cladogram showing the relationships between taxa at different taxonomic levels. Each circle represents a hierarchical structure, followed by phylum, class, order, family and genus. Different phyla are marked with different colors. The size of the nodes represents the abundance of the taxon. **(C)** Linear discriminant analysis (LDA) scores with the LEfSe tool for taxa, with LDA scores >4 and *p* < 0.05 shown in the histogram.

To investigate taxonomic differences between NAFL and NASH groups at the phylum and genus levels, we employed edgeR and LEFSe for analysis. The relative abundance of bacteria was assessed across all three groups at both the phylum and genus levels, revealing statistically significant disparities. At the phylum level, each sample exhibited distinct species composition (Figure 3A), with Bacteroidetes, Firmicutes, Proteobacteria, Fusobacteria, and Actinobacteria being the predominant phyla accounting for 95% or more abundance in all three groups (Figure 3B). At the phylum level, the predominant bacteria in the NAFL group were predominantly members of the phylum Bacteroidetes (73.9%), Firmicutes (17.2%), Fusobacteria (3.4%), and Proteobacteria (4.6%). The NASH group was also dominated by members of Bacteroidetes (71%), Firmicutes (19.7%), Fusobacteria (4.1%), and Proteobacteria (4.8%). At the genus level, Bacteroides, Prevotella_9, Faecalibacterium, Megamonas, and Fusobacterium were the predominant genera accounting for 90% or more abundance in all three groups (Figure 3C). The results of the edgeR package analysis, illustrated by the Manhattan plot (Figure 4A) showed that there was an enrichment of Bacteroidetes and Fusobacteria, while the ratios of Firmicutes and Proteobacteria were depleted in both the NAFL group and NASH group, as depicted in Supplementary Figure S2. Compared to the NAFL group, the NASH group exhibited higher levels of Firmicutes but lower levels of Bacteroidetes at phylum level, with Bacteroides being enriched as the most abundant genus (FDR < 0.05; Supplementary Figure S2). The relative abundance of Bacteroides and Fusobacterium was significantly higher in the NAFL group, while Prevotella_9 was lower compared to the control group. In the NASH group, there was an enrichment of Megamonas and Fusobacterium. Furthermore, the NASH group exhibited higher levels of Megamonas and Fusobacterium compared to the NAFL group.

The LEfSe analysis was employed to construct a cladogram for the identification of specific bacteria associated with the three groups (Figure 4B). Megamonas and Fusobacterium were found to be significantly enriched in the fecal samples of patients belonging to the NASH group, as indicated by high LDA scores (log10 > 4). Conversely, Subdoligranulum and Ruminococcus_2 were identified as the predominant microbiota in the control group (Figure 4C).

### 3.4 Difference analysis of intestinal flora expression between NAFL and NASH patients

**Figure 5:**
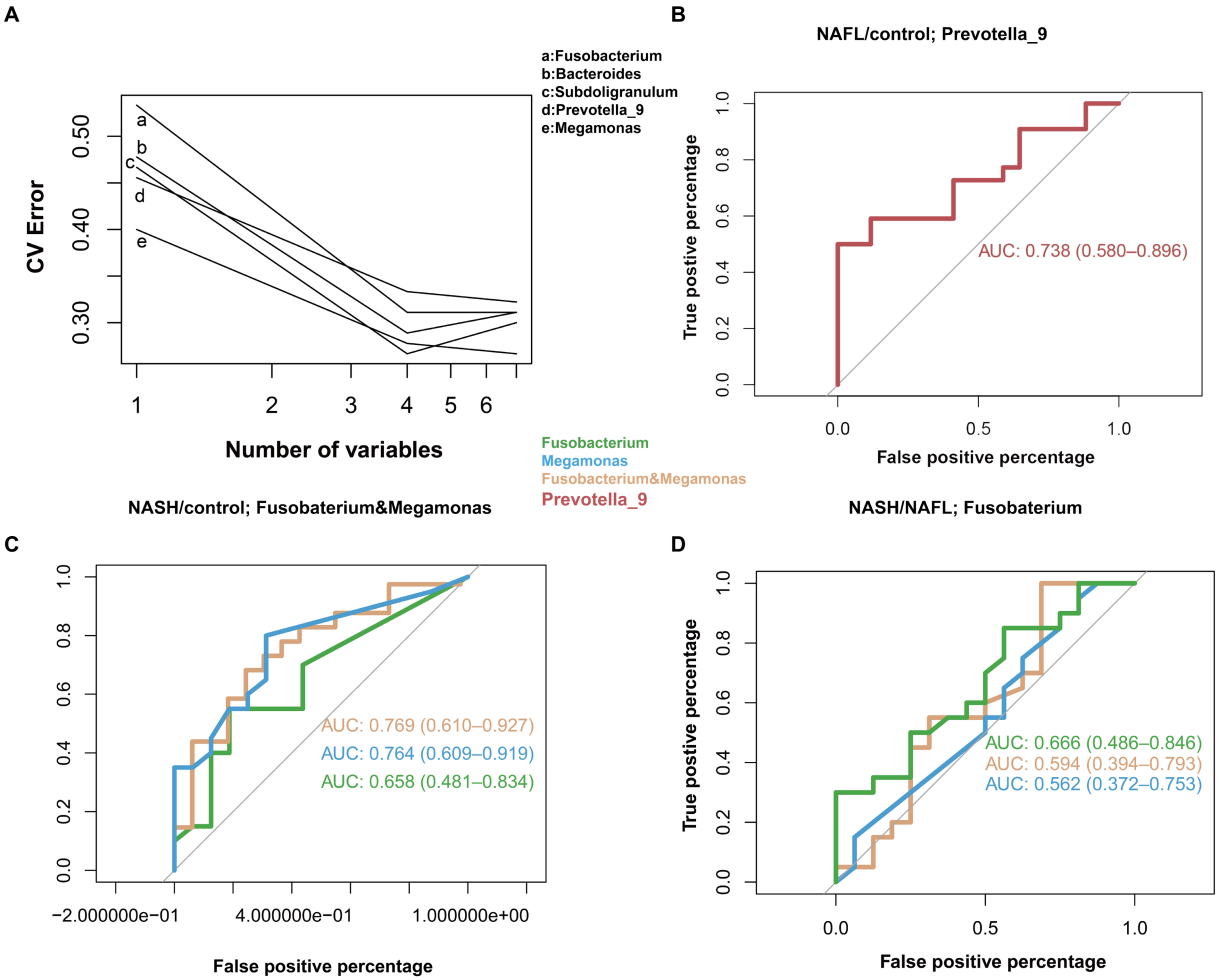
Diagnostic potential of gut microbial markers in NAFL and NASH patients. Diagnostic potential of gut microbial markers in NAFL and NASH patients. **(A)** Five microbial markers (Bacteroides, Prevotella_9, Ruminococcus_2, Subdoligranulum, Megamonas, Fusobacterium) were selected as the markers set by the random forest model. **(B)** The classification performance of the multivariable logistic regression model using relative abundance of genera (Prevotella_9) was assessed using area under the ROC. The AUC value of 0.738 with 95% CI of 0.580–0.896 between control versus NAFL group. **(C)** Classification performance of a multivariable logistic regression model using the relative abundance of genera (Megamonas, Fusobacterium, and a combination of both) was evaluated using the area under the ROC. The highest AUC value between the control group and the NASH group was 0.769, with a 95% CI of 0.610–0.927, belonging to the combination of Megamonas and Fusobacterium. **(D)** Classification performance of a multivariable logistic regression model using the relative abundance of genera (Megamonas, Fusobacteria, and a combination of both) evaluated using the area under the ROC. The highest AUC value between the NAFL and NASH groups was 0.666, 95% CI, belonging to the genus Fusobacterium.

In the study cohort, we constructed a random forest classifier model for 65 healthy controls, 64 NAFL and 53 NASH to evaluate the diagnostic efficacy of intestinal microbiome markers and explore their potential as a non-invasive diagnostic tool for NAFL and NASH. Through a 10-fold cross-validation of the random forest model, the dataset was partitioned into 10 subsets, with nine of them utilized for training purposes and one reserved for testing. The accuracy rate was computed for each experiment, and the average accuracy rate from 10 iterations serves as an estimation of the algorithm’s performance. This process was repeated using 10-fold cross-validation to obtain a final averaged estimation of the algorithm’s accuracy. Using this approach, the first five microbial genera were selected as the best marker set for NAFL and NASH (Figure 5A), Combining the results of species abundance and LEfSe analyses, we decided to assess the potential value of Prevotella_9, Megamonas and Fusobacterium as biomarkers. The results showed that compared with the control group, Prevotella_9 distinguished NAFL with an AUC of 0.738 (95% CI = 0.580–0.896; Figure 5B). However, the expression of Megamonas and Fusobacterium continues to progressively increase in the control group, NAFL group, and NASH group, with a particularly significant elevation observed in the NASH group. The combined presence of these taxa effectively distinguishes NASH with an AUC of 0.769 (95% CI = 0.610–0.927; Figure 5C). It is evident that Prevotella_9 exhibits a significant decrease in expression as the most representative bacterial taxon in NAFL, while Megamonas and Fusobacterium demonstrate a notable increase in expression as the most representative taxa in NASH. To differentiate between the microflora profiles of the NAFL and NASH groups, a simple forest analysis was conducted using single and combined data for Megamonas and Fusobacterium compared to those solely of the NAFL group (Figure 5D), this yielded an AUC value of 0.666 for single Fusobacterium alone. These findings suggest a potential correlation between elevated levels of Megamonas and Fusobacterium and disease progression.

### 3.5 Public data validation

To validate the robustness and generalizability of the analysis findings, we selected two sets of publicly available NAFLD-related sequence data for species diversity analysis. The respective Project ids associated with these datasets are PRJNA737039 and PRJNA540738. Upon comparing our data with the two public datasets, it is evident that the composition and variability of gut microbiota across different countries and regions are substantial. These discrepancies are likely influenced by factors such as geographical environment and dietary practices. Notably, our analysis results exhibit a higher degree of similarity with the PRJNA737039 project, with predominant species including Bacteroides, Prevotella_9, Faecalibacterium, and Roseburia. The observed differences in species composition and significant disparities may be attributed to disease classification standards, regional influences, and other variables. This suggests that the findings of our research may have greater relevance and applicability within the context of China (Supplementary Figure S3). The PRJNA737039 dataset originated from China, while the PRJNA540738 dataset was obtained from Germany. Combining species diversity analysis with LEfSe difference analysis revealed that in Project PRJNA737039, Bacteroidetes, Prevotella_9 and Faecalibacterium were significantly expressed in both NAFL and NASH groups compared to healthy controls, which is consistent with the species composition observed in our own sample. In contrast, Project PRJNA540738 exhibited a distinct pattern of species expression, including Bacteroides, Blautia, Bacillus, and Faecalibacterium. Our analysis findings exhibit a high degree of resemblance to the taxonomic composition of the Chinese dataset, while displaying significant disparities with the European data. This underscores the credibility of our analytical results and further highlights dissimilarities in gut microbiome composition between Asian and European NAFLD patients, potentially attributed to factors such as dietary habits and living environment.

### 3.6 Functional analysis of the gut microbiome

The Bugbase platform was utilized to predict the corresponding characteristics and content of each bacterium within the three groups. The compositional differences of aerobic bacteria, anaerobic bacteria, facultative anaerobes, mobile element carriers, biofilm formers, gram-negative species, gram-positive species, potentially pathogenic strains, and stress-tolerant organisms in these three groups are illustrated in Supplementary Figure S4. Bacteroides exhibited higher enrichment in the NAFL group while Firmicutes were enriched in the NASH group with regards to their high-level phenotypes present potential pathogenicity (Supplementary Figure S4H).

**Figure 6:**
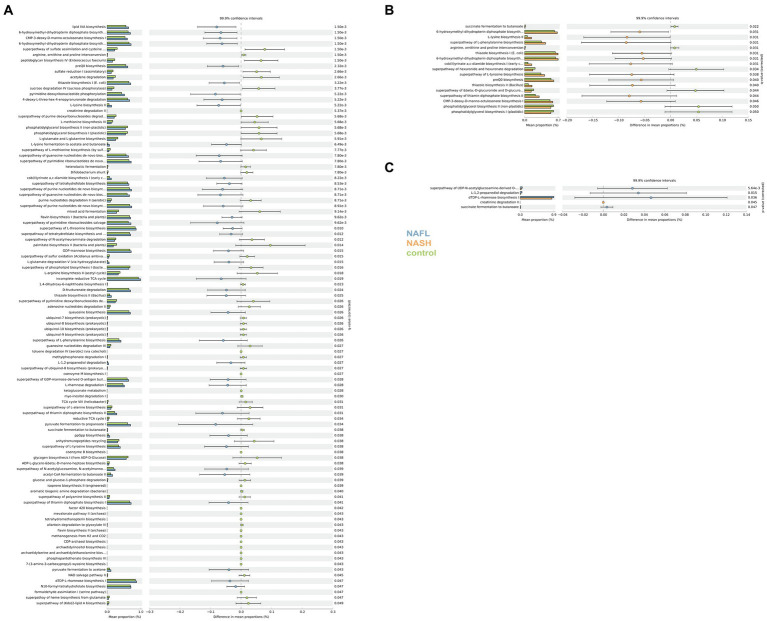
Functional analysis of the predicted metagenomes. **(A)** Difference in relative abundance of predicted microbial genes related to metabolism information between NAFLD and healthy controls. **(B)** Difference in relative abundance of predicted microbial genes related to metabolism information between NASH and healthy controls. **(C)** Difference in relative abundance of predicted microbial genes related to metabolism information between NASH and NAFLD. Data were processed through PICRUSt2’s 16S rRNA sequencing data using level 3 of the Kyoto Encyclopedia of Genes and Genomes (KEGG) orthologues and using a Student’s t-test. The significance of gut microbial species derived from blocked two-sided Wilcoxon tests (+indicates statistical significance FDR < 0.05, 99.9% confidence intervals).

PICRUSt and PICRUt2 were utilized for predictive functional analysis. KEGG pathway comparisons were conducted to investigate potential variations in the functional composition of the microbiome among NAFL, NASH, and control groups. PICRUST2 essentially encompasses the findings obtained from PICRUST (Supplementary Figure S5). In comparison to the control group, numerous pathway differences were observed in NAFL, such as increased abundance of lipid IVA biosynthesis, 6-hydroxymethyl-dihydropterin diphosphate biosynthesis I, CMP-3-deoxy-D-manno-octulosonate biosynthesis I, and 6-hydroxymethyl-dihydropterin diphosphate biosynthesis III (Chlamydia), while decreased abundance was found in superpathway of purine deoxyribonucleosides degradation, L-methionine biosynthesis III, and phosphatidylglycerol biosynthesis I (plastidic; Figure 6A). The pathways were significantly different in the NASH group compared with the control group. Specifically, 6-hydroxymethyl-dihydropterin diphosphate biosynthesis, L-lysine biosynthesis II, and superpathway of L-phenylalanine biosynthesis were more abundant in NASH while superpathway of hexuronide and hexuronate degradation, phosphatidylglycerol biosynthesis II (non-plastidic), and phosphatidylglycerol biosynthesis I (plastidic) was less abundant (Figure 6B). Compared to the control group, there were significant differences in pathways between NASH and NAFL groups. Creatinine degradation II was more abundant in NASH compared to NAFL while superpathway of UDP-N-acetylglucosamine-derived O-antigen building blocks biosynthesis, L-1,2-propanediol degradation, dTDP-L-rhamnose biosynthesis I, succinate fermentation to butanoate, and Flavone and flavonol biosynthesis were significantly less abundant than the NAFL group (Figure 6C).

## 4 Discussion

There is already evidence to suggest that the development of fatty liver is related to gut bacteria, including dysbiosis-induced dysfunction of the intestinal epithelial barrier. This dysfunction allows the translocation of bacterial components and leads to liver inflammation. Additionally, various metabolites produced by the gut microbiota may affect the liver, thereby regulating susceptibility to NAFLD.

In our study, 16S amplicon analysis revealed alterations in the gut microbiota composition in NAFL and NASH diseases compared to a healthy control group, as well as differences between the two diseases. The NAFL group exhibited an increased abundance of Gram-negative bacteria and a decreased abundance of Gram-positive bacteria compared to the control group. Bacterial diversity showed significant differences (*p* < 0.05) among the three groups, with species diversity progressively decreasing by threefold in the NASH group. This shift toward specific bacterial types promotes an increase in pathogenic abundance (Shen et al., 2017). At the phylum level, the abundances of Bacteroidetes and Fusobacteria, were higher in NAFL and NASH patients than in the control group, while the abundances of firmicutes were lower. At the genera level, a significant reduction in the abundance of Prevotella was observed in the NAFL group. In contrast, the NASH group showed a higher proportion of Megamonas and Fusobacterium (*p* < 0.05). These findings align with existing data from Asian patients but differ substantially from European case data, suggesting variations in gut microbiome composition between Eurasian populations (Quesada-Vazquez et al., 2020).

Through the analysis of three main bacterial genera, namely Prevotella_9, Megamonas, and Fusobacterium, it was observed that compared to the control group, Prevotella_9 exhibited a discriminative ability for distinguishing NAFL with an AUC of 0.738 (95% CI = 0.580–0.896; Figure 5B). Conversely, the combination of Megamonas and Fusobacterium demonstrated a discriminative potential for identifying NASH with an AUC of 0.769 (95% CI = 0.610–0.927; Figure 5C). In the NAFL group, we observed that Fusobacterium alone had a superior diagnostic performance than Megamonas alone, or Fusobacterium and Megamonas combined. More specifically, as shown in Figure 5D, the AUC of Fusobacterium is 0.666 (95% CI = 0.486–0.846), surpassing that of Megamonas alone (0.562, 95% CI = 0.372–0.753), or that of Fusobacterium and Megamonas combined (0.594, 95% CI = 0.394–0.793). These observational results suggest that an increased abundance of Prevotella_9 has diagnostic value for NAFL, while elevated levels of Megamonas and Fusobacterium may indicate a more severe disease state that has progressed to the NASH stage, especially the increase in Fusobacterium, which has more significant diagnostic meaning. In other words, changes in these three main gut genera could provide a non-invasive basis for diagnosing different stages of NAFLD.

Building upon previous research, we conducted an analysis on the potential pathogenic mechanisms of Bacteroides and Fusobacterium. The gut microbiota has the ability to ferment dietary carbohydrates into ethanol, which subsequently enters the bloodstream and is eventually metabolized by the liver. Notably, Bacteroides is recognized as one of the primary producers of ethanol (Amaretti et al., 2007). Both animal studies and clinical trials have consistently demonstrated that NAFL patients exhibit significantly elevated levels of gastrointestinal ethanol compared to control groups, with a strong correlation observed between these levels and alterations in gut microbiota composition (Cope, 2000). The higher abundance of Bacteroides in the NAFL group, compared to the control group, implies that an elevated Bacteroides content may contribute to liver damage by promoting endogenous ethanol production. Fusobacterium has been reported to produce short-chain fatty acids with both anti-inflammatory and pro-inflammatory properties (Fan et al., 2019). An increase in Fusobacterium abundance can serve as a marker for tumor occurrence and intestinal inflammation (Kostic et al., 2012; Zhang et al., 2018). Fusobacterium, a pro-inflammatory bacterium known to impair the integrity of the intestinal barrier (Neubauer et al., 2019), has been observed in fatty samples and is associated with the exacerbation of liver steatosis and recurrence of NAFL (Fan et al., 2019). The Prevotella spp. and P. Capri complexes are commonly associated with non-Western dietary patterns characterized by a high intake of carbohydrates, resistant starch, and fiber content. There is a negative correlation between the relative abundance of Prevotella and Bacteroides (Carelli et al., 2023).

Prevotella has a number of classification methods, and it has been reported that a 16S rRNA sequence method was utilized to reclassify Prevotella and identify 5 genera (Adkins et al., 2017). This study unveiled a negative correlation between changes in Prevotella-9 abundance and Bacteroides, which aligns with previous research findings. Previous studies have reported diverse roles for Prevotella in gut health, while P. Capri has demonstrated potential benefits in glucose homeostasis and host metabolism. Conversely, another study identified an association between P. Capri and insulin resistance. This study revealed a significant decrease in NAFLD when comparing levels of Prevotella 9 between healthy individuals and those at the NAFL stage, suggesting that reduced Prevotella abundance may contribute to impaired carbohydrate and starch metabolism, both of which are linked to obesity and steatosis. The impact of Prevotella on human health remains uncertain, leading to ongoing debates regarding its potential benefits or harms. However, it is undeniable that the enzymatic digestion of polysaccharides within the Prevotella library plays a crucial role in digestive dynamics and intestinal homeostasis. It should be noted that the relationship between Prevotella and inflammation, particularly NASH, still lacks clarity. In this study, although there was some recovery in Prevotella abundance from NAFL to NASH stages, it remained lower than that observed in healthy individuals. The relationship between the inflammatory response of NAFL and the progression of NASH, as well as fibrosis, remains unclear. Some studies have reported a significant increase in Prevotella abundance in NASH with fibrosis (Boursier et al., 2016). Compared to the control group, the NASH group exhibited higher abundances of Megamonas and Fusobacterium. Megamonas, a core gut bacterium, may be indicative of Asian ethnicities. It is closely associated with diseases such as inflammatory bowel disease, colorectal cancer, ankylosing spondylitis (AS), autism spectrum disorder (ASD), and obesity. Previous studies on the gut microbiota of European subjects did not report dominant presence of the genus Megamonas; it was only observed in studies conducted on Chinese individuals, suggesting its potential characteristic nature within the Asian population (Boursier et al., 2016). Studies have revealed a negative correlation between the abundance of Megamonas and the rate of weight loss, exhibiting a significant increase in the obese cohort. The average BMI of our study population exceeded 29 kg/m^2, with nearly all NASH patients displaying abnormal ALT levels. These findings suggest a substantial elevation in Megamonas abundance within the Chinese obese population, which, when combined with an augmentation in Fusobacterium abundance, further compromises intestinal barrier integrity and facilitates the progression from NAFL to NASH.

To further investigate the impact of dysbiosis on microbial metabolism, we utilized gene transcription analysis techniques. KEGG pathway analysis revealed significant alterations in metabolic pathways associated with NAFLD, including notable variations in transcription, immune system disorders, signaling molecules and their interactions, environmental adaptation, transport and catabolic metabolism. The changes of these metabolites are correlated with disease progression. In comparison to the NAFL group, the NASH group exhibited significant differences in all aspects of glucose metabolism as well as biosynthesis of flavonoids and flavonols.

In the KEGG predicted functional pathway analysis of both the NASH and NAFL groups, significant increases were observed in the biosynthesis of flavones and flavonols, glucose metabolism pathways (including the biosynthesis superpathway of O-antigen building blocks derived from UDP-N-acetylgalactosamine), degradation pathways of L-rhamnose and L-fucopyranose (including L-1,2-propanediol degradation), biosynthesis of dTDP-L-rhamnose I, and butyrate fermentation from succinate in the NAFL group. Additionally, creatinine degradation II was significantly higher in the NASH group. It has been reported that flavonoid compounds are representative substances that protect liver function by regulating CYP2E19 (Liu et al., 2021). Compared to NAFL, the expression of flavones and flavonol biosynthesis pathways is reduced in NASH. Numerous studies have demonstrated that flavonoid compounds exert physiological functions such as antioxidation, lipid-lowering, blood sugar regulation, and inflammation inhibition by modulating the gut microbiota, thereby preventing various diseases. The presence of an inflammatory response in NASH and may be associated with alterations in this pathway. Research has reported the potential role of ferritin in reflecting the inflammatory state associated with NAFLD and its progression, with elevated ferritin levels indicating inflammation and liver damage (Shah and Kowdley, 2019). Although we did not initially focus on inflammatory biomarkers such as ferritin in our study, in future research on the inflammation associated with NASH, we can consider measuring inflammatory biomarkers like ferritin in the serum of patients with NAFLD or NASH. Differences in various carbohydrate metabolisms also indicate changes in the fundamental metabolism between the two groups (Sandoval et al., 2020; Kariagina and Doseff, 2022; Tan et al., 2022). This finding provides support for the hypothesis that NAFLD is fundamentally a metabolic disorder. Our study focused on the Chinese population, with participants from various regions of China, including Shenzhen, a city known for its immigrant population. It should be noted that the relationship between gut microbiome dysbiosis and NAFLD disease cannot be extrapolated to European and American populations. Additionally, it is important to acknowledge that the use of 16S rRNA gene sequencing methods has inherent limitations in accurately determining the composition of the microbiome.

## 5 Conclusion

In summary, this study employed 16S amplicon analysis to uncover changes in the composition of gut microbiota during the progression of NAFLD, characterized by a gradual decrease in species diversity. At the phylum level, significant shifts were observed in the abundance of key bacterial groups including Bacteroidetes, Firmicutes, Fusobacteria. Moreover, at the genus level, a noticeable reduction was observed abundance of Prevotella within the NAFL group while an increased proportion of Megamonas and Fusobacterium was evident in the NASH group. The prominent expression of Megamonas and Fusobacterium can serve as a valuable biological marker for identifying NASH. KEGG pathway analysis revealed significant disruptions in various glucose metabolism pathways within the NASH group compared to the mild NAFL group, along with a notable deficiency in flavones and flavonols biosynthesis. Our study has elucidated significant associations between gut microbiome alterations and the progression from NAFL to NASH in patients with NAFLD. The differential microbial profiles observed in NASH patients underscore the potential of microbiota-based biomarkers for non-invasive diagnosis, which could mitigate the reliance on liver biopsies and enable earlier detection and intervention. Our findings suggest that certain bacteria could serve as targets for microbiome-modulating therapies, such as Megamonas and Fusobacterium, opening new avenues for precision medicine in NAFLD management. Moreover, the novel takes identified in our study invite further investigation into their functional roles in NAFLD pathophysiology. The potential for microbiome-based interventions to attenuate or reverse NAFLD progression is an exciting prospect, but it is one that must be approached with rigorously designed clinical trials.

## Data availability statement

The datasets analyzed in this study can be found in the China National GeneBank (CNGB) with a program ID CNP0003220.

## Ethics statement

The studies involving humans were approved by Ethics Committee of Shenzhen Hospital of Traditional Chinese Medicine (ethical approval number: K2017-024). The studies were conducted in accordance with the local legislation and institutional requirements. Written informed consent for participation in this study was provided by the participants’ legal guardians/next of kin.

## Author contributions

FH: Data curation, Writing – original draft. BL: Software, Writing – original draft. FX: Writing – original draft. YX: Data curation, Writing – review & editing. ZH: Data curation, Writing – review & editing. JL: Data curation, Writing – review & editing. JM: Software, Writing – review & editing. YZ: Software, Writing – review & editing. HZ: Software, Writing – review & editing. ZX: Software, Writing – review & editing. PG: Software, Writing – review & editing. YL: Funding acquisition, Writing – review & editing. LB: Funding acquisition, Writing – review & editing. GT: Funding acquisition, Writing – review & editing. XF: Funding acquisition, Writing – original draft. FL: Writing – review & editing.
