# Supplementary material for: From gut to liver: unveiling the differences of intestinal microbiota in NAFL and NASH patients
Source: Front Microbiol. 2024 Apr 4;15:1366744. doi: 10.3389/fmicb.2024.1366744 (PMC11024258; doi:10.3389/fmicb.2024.1366744)
Supplement: Supplementary file 2 [file Presentation_1.PDF]

Supplementary Material

Supplementary Figures S1-5

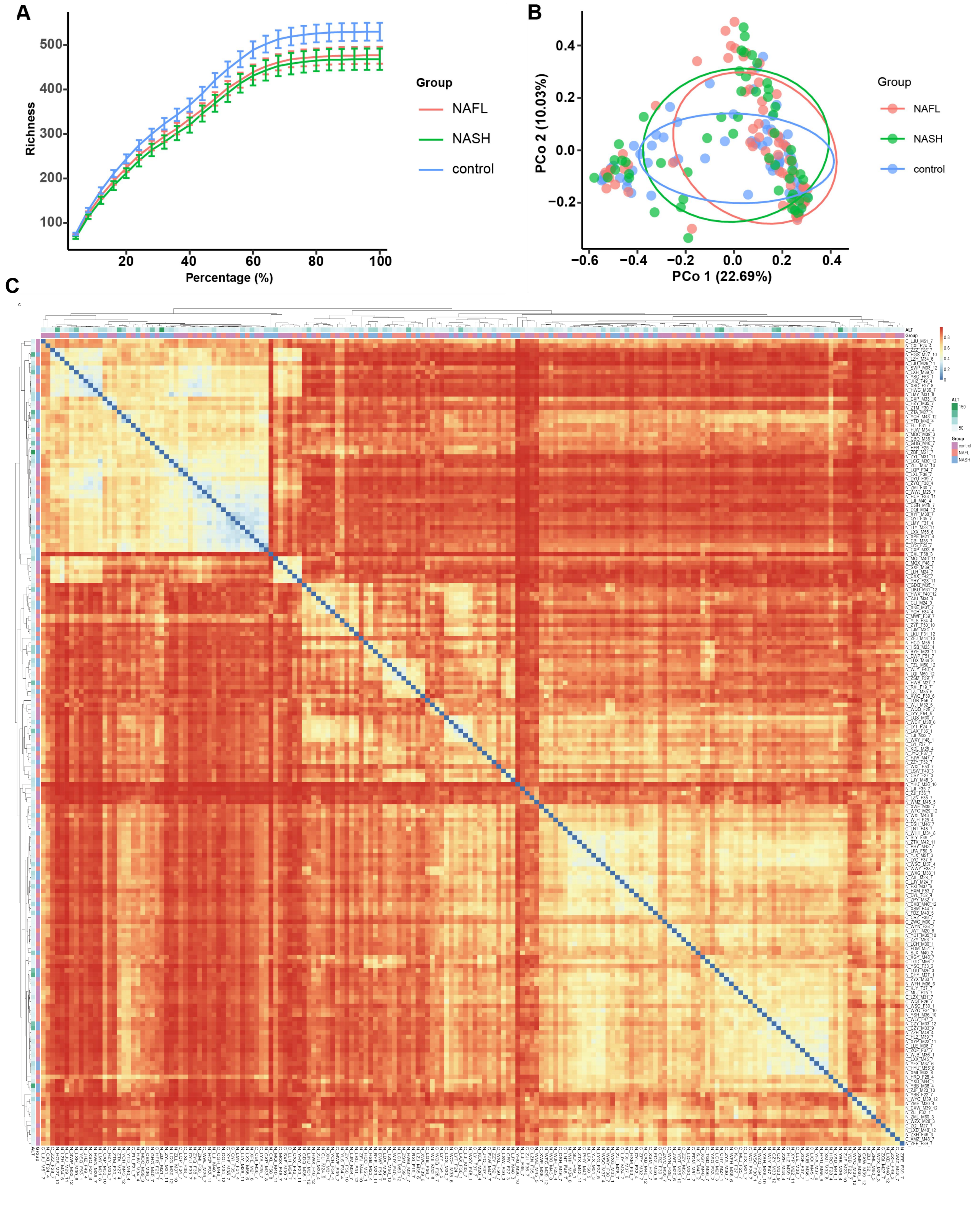

Figure S1 Supplementary figure for diversity analysis of three groups

(A) A rarefaction curve between the number of OTUs and the number of sequences in control, NAFL and NASH group. (B) Principal coordinate analysis (PCoA) plots of bacterial beta diversity of the three groups based on Bray-Curtis dissimilarity. (C) A heatmap showed the relative abundance and distribution of the OTUs among all samples of the three groups.

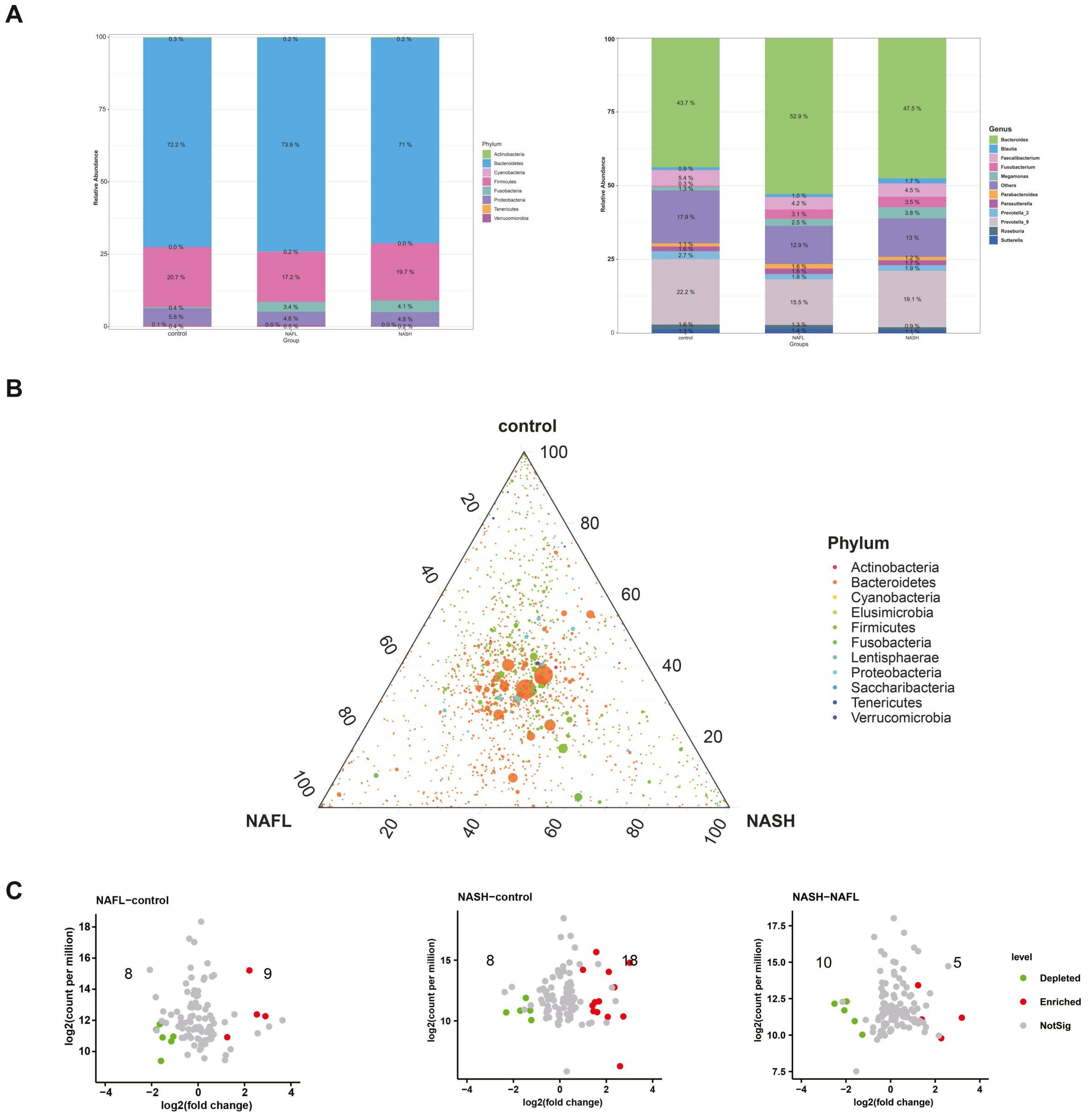

Figure S2 Comparison of species composition and differences among the three groups

(A) Comparisons of the taxonomic classifications of control, NAFL, NASH group, showed the percentage of each bacteria at the phylum and genus levels. (B) The ternary diagram showed the overall differences in Top 11 phyla composition among the three groups. (C) The volcano plot showed the fold difference and relative abundance of OTU between groups, from left to right, NAFL and control, NASH and control, NAFL and NASH were comparison ($p<0.01$).

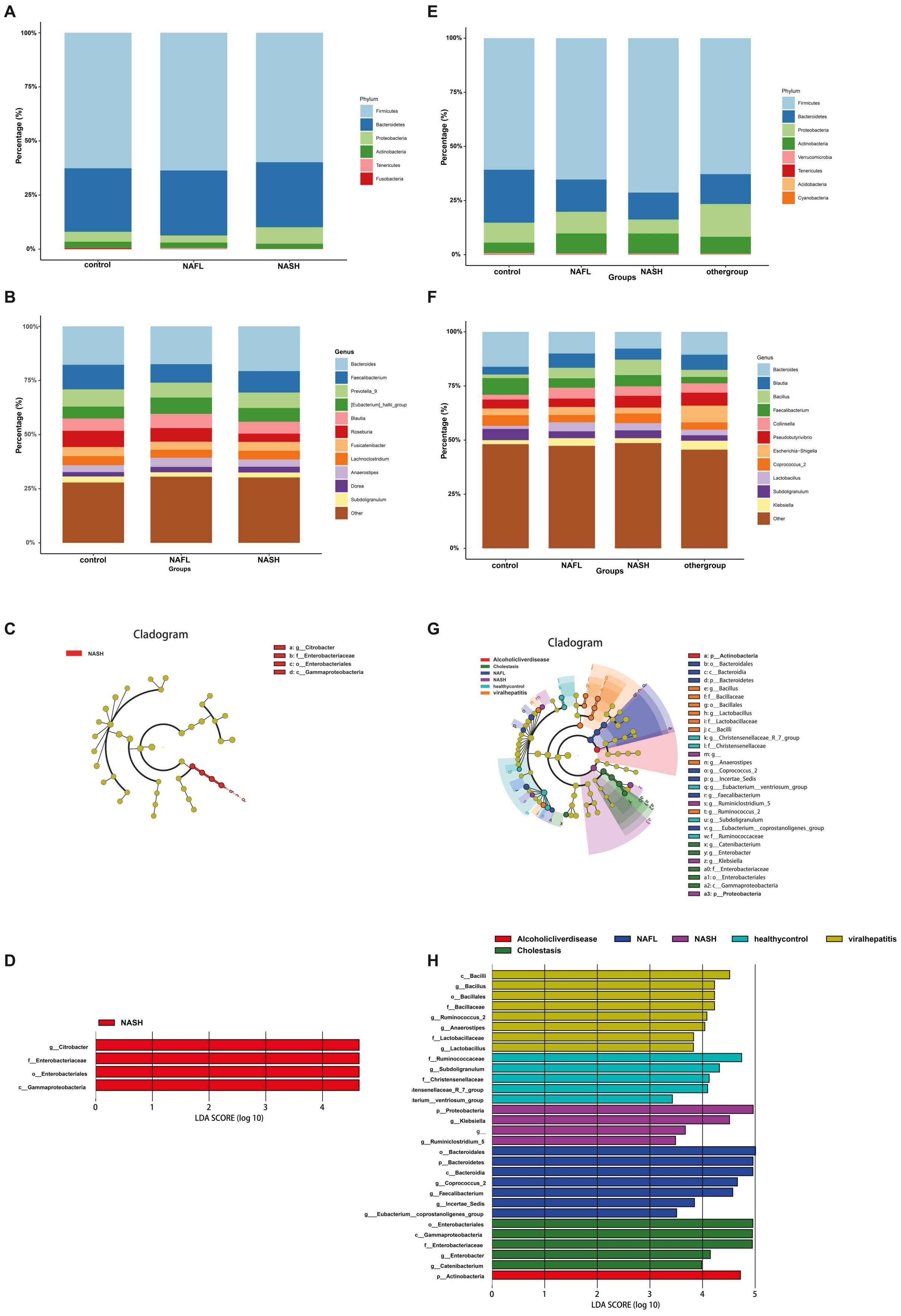
A
100%
75%
50%
25%
0%
Percentage (%)
control
NAFL
NASH
Phylum
Firmicutes
Bacteroidetes
Proteobacteria
Actinobacteria
Tenericutes
Fusobacteria
E
Groups
othergroup
Verrucomicrobia
Acidobacteria
Cyanobacteria
B
F
Genus
Bacteroides
Faecalibacterium
Prevotella_9
[Eubacterium]_hallii_group
Blautia
Roseburia
Fusicatenibacter
Lachnoclostridium
Anaerostipes
Dorea
Subdoligranulum
Other
Bacillus
Collinsella
Pseudobutyrivibrio
Escherichia−Shigella
Coprococcus_2
Lactobacillus
Klebsiella
C
Cladogram
NASH
a: g__Citrobacter
b: f__Enterobacteriaceae
c: o__Enterobacteriales
d: c__Gammaproteobacteria
G
Cladogram
Alcoholicliverdisease
Cholestasis
NAFL
NASH
healthycontrol
viralhepatitis
a: p__Actinobacteria
b: o__Bacteroidales
c: c__Bacteroidia
d: p__Bacteroidetes
e: g__Bacillus
f: f__Bacillaceae
g: o__Bacillales
h: g__Lactobacillus
i: f__Lactobacillaceae
j: c__Bacilli
k: g__Christensenellaceae_R_7_group
l: f__Christensenellaceae
m: g__
n: g__Anaerostipes
o: g__Coprococcus_2
p: g__Incertae_Sedis
q: g___Eubacterium__ventriosum_group
r: g__Faecalibacterium
s: g__Ruminiclostridium_5
t: g__Ruminococcus_2
u: g__Subdoligranulum
v: g___Eubacterium__coprostanoligenes_group
w: f__Ruminococcaceae
x: g__Catenibacterium
y: g__Enterobacter
z: g__Klebsiella
a0: f__Enterobacteriaceae
a1: o__Enterobacteriales
a2: c__Gammaproteobacteria
a3: p__Proteobacteria
D
NASH
g__Citrobacter
f__Enterobacteriaceae
o__Enterobacteriales
c__Gammaproteobacteria
0 1 2 3 4
LDA SCORE (log 10)
H
Alcoholicliverdisease
NAFL
NASH
healthycontrol
viralhepatitis
Cholestasis
c__Bacilli
g__Bacillus
o__Bacillales
f__Bacillaceae
g__Ruminococcus_2
g__Anaerostipes
f__Lactobacillaceae
g__Lactobacillus
f__Ruminococcaceae
g__Subdoligranulum
f__Christensenellaceae
stensenellaceae_R_7_group
terium__ventriosum_group
p__Proteobacteria
g__Klebsiella
g__
g__Ruminiclostridium_5
o__Bacteroidales
p__Bacteroidetes
c__Bacteroidia
g__Coprococcus_2
g__Faecalibacterium
g__Incertae_Sedis
g___Eubacterium__coprostanoligenes_group
o__Enterobacteriales
c__Gammaproteobacteria
f__Enterobacteriaceae
g__Enterobacter
g__Catenibacterium
p__Actinobacteria
0 1 2 3 4 5
LDA SCORE (log 10)

Figure S3 Species composition of two sets of public data

A,B,C,D

~~(A)Taxonomic composition in public data named project PRJNA737039.~~

~~(B)Taxonomic composition in public data named project PRJNA540738.~~

Samples A, B, C, and D originate from project PRJNA737039, with datasets contributed by China. Conversely, samples E, F, G, and H are sourced from project PRJNA540738, featuring data collected from Germany. (A) Phyla composition for PRJNA737039. (B) Genera composition for PRJNA737039. (C) Cladogram showing the relationships between taxa at different taxonomic levels, with data from project PRJNA737039, China. (D) Linear discriminant analysis (LDA) scores with the LEfSe tool for taxa, with LDA scores >4 and $p < 0.05$ shown in the histogram, with data from project PRJNA737039, China. (E) Phyla composition for PRJNA540738. (F) Genera composition for PRJNA540738. (G) Cladogram showing the relationships between taxa at different taxonomic levels, with data from project PRJNA540738,Germany. Each circle represents a hierarchical structure, followed by phylum, class, order, family and genus. Different phyla are marked with different colors. The size of the nodes represents the abundance of the taxon. (H) Linear discriminant analysis (LDA) scores with the LEfSe tool for taxa, with LDA scores >4 and $p < 0.05$ shown in the histogram. with data from project PRJNA540738,Germany. Upon comparing the two public datasets, it is evident that the composition and variance of gut microbiota across different countries and regions are substantial. These discrepancies are likely influenced by factors such as geographical environment and dietary practices. Notably, our analysis results exhibit a higher degree of similarity with the PRJNA737039 project, with predominant species including Bacteroides, Prevotella_9, Faecalibacterium, and Roseburia. The observed differences in species composition and significant disparities may be attributed to disease classification standards, regional influences, and other variables. This suggests that the findings of our research may have greater relevance and applicability within the context of China.

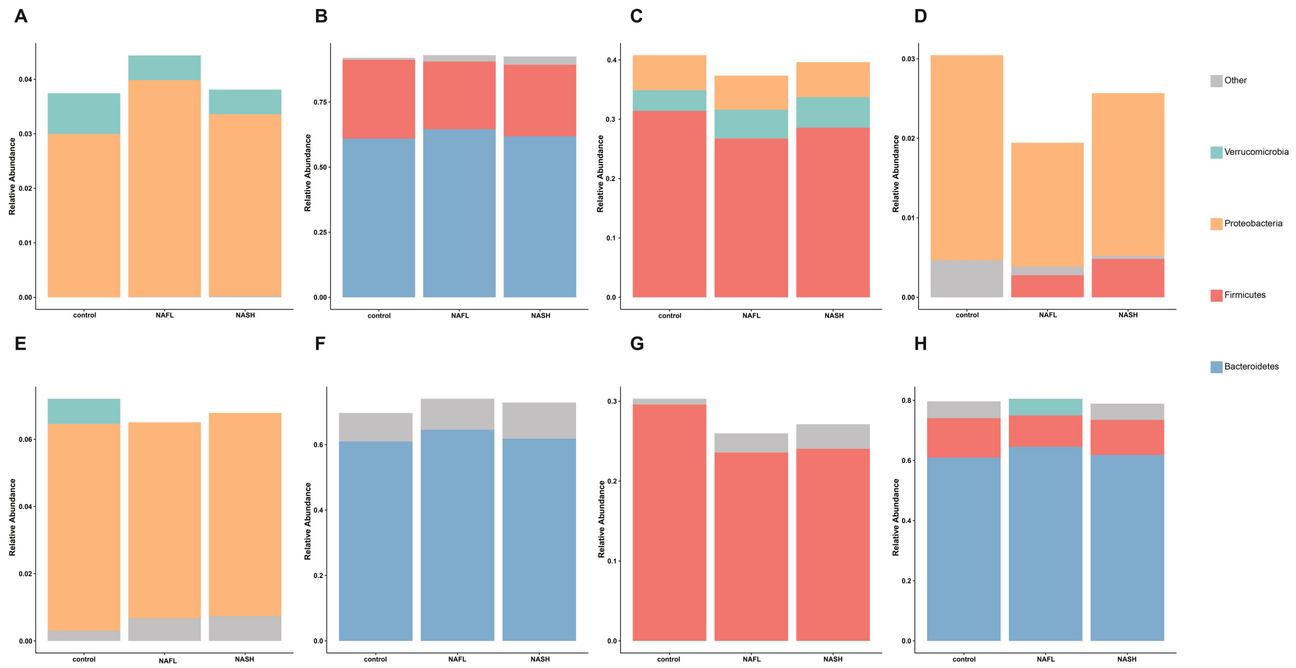

Figure S4 Bugbase analysis predicts gut microbiome species categories and characteristics

The composition differences of (A) aerobic bacteria, (B) anaerobic bacteria, (C) Facultative anaerobic, (D) Contains Mobile Elements, (E) Forms Biofilms, (F) Gram Negative, (G) Gram Positive, (H) Potentially Pathogenic in the three groups.

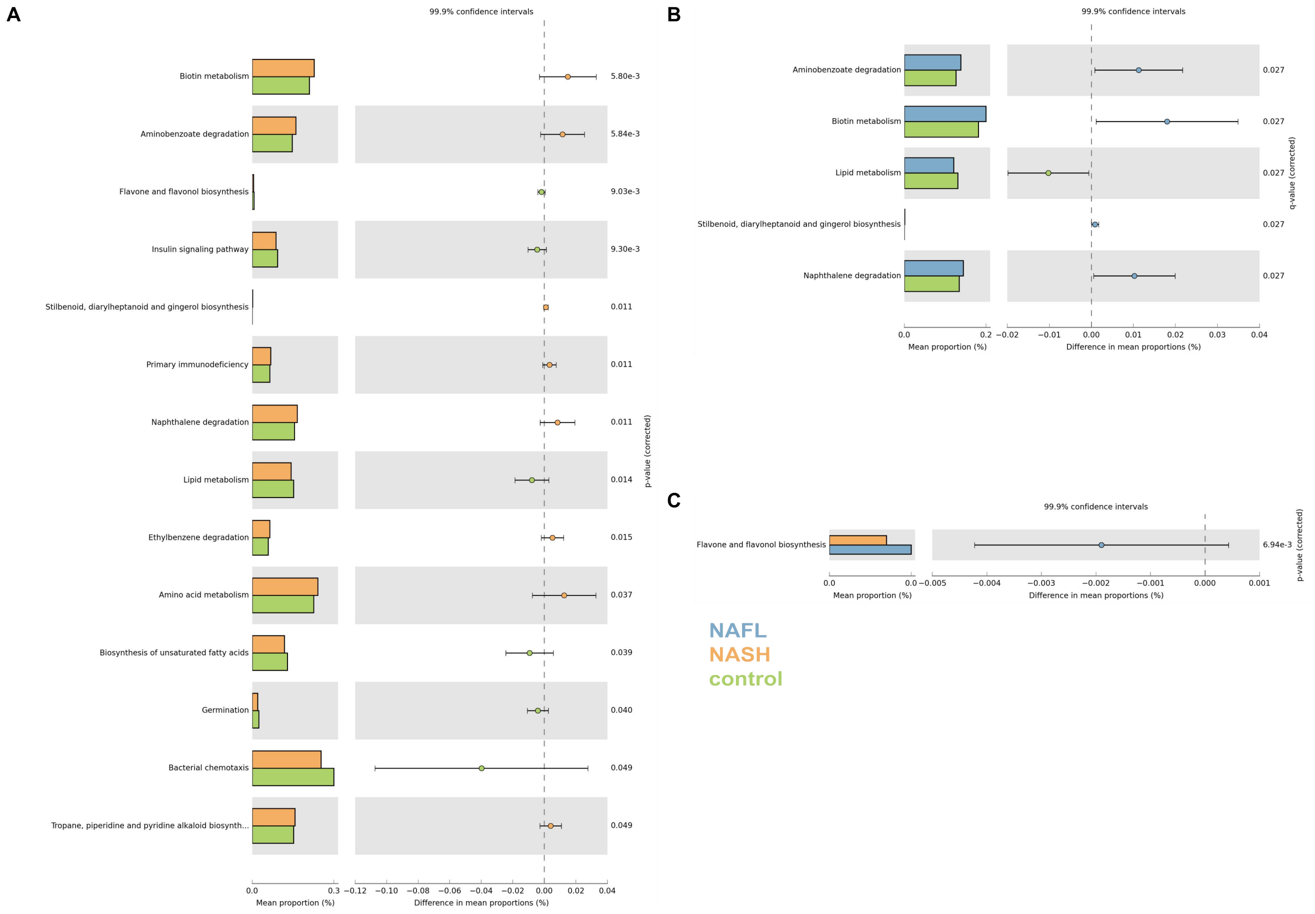

Figure S5 Phylogenetic Investigation of Communities by Reconstruction of Unobserved States analysis predicts the metabolic functional profile of gut microbiome

(A) Difference in relative abundance of predicted microbial genes related to metabolism information between NAFL and healthy controls. (B) Difference in relative abundance of predicted microbial genes related to metabolism information between NASH and healthy controls. (C) Difference in relative abundance of predicted microbial genes related to metabolism information between NASH and NAFL. Data were processed through PICRUSt's 16S rRNA sequencing data using level 3 of the Kyoto Encyclopedia of Genes and Genomes (KEGG) orthologues and using a Student's t-test. The significance of gut microbial species derived from blocked two-sided Wilcoxon tests (+ indicates statistical significance FDR < 0.05, 99.9% confidence intervals)
